# The Eighth Periodical International Ophthalmological Congress

**Published:** 1895-04

**Authors:** Alvin A. Hubbell

**Affiliations:** Buffalo, N. Y.


### THE EIGHTH PERIODICAL INTERNATIONAL OPHTHALMOLOGICAL CONGRESS, HELD AT EDINBURGH, AUGUST 7–11, 1894.

By ALVIN A. HUBBELL, M. D., Buffalo, N. Y.

(*Concluded from page 487.*)

### SUPPLEMENTARY REPORT.

Since my report of the congress was completed, the official transactions have been received. These include other communications which were presented to the congress, but not read, and they are of so much importance that abstracts of them are hereby appended.

### TITLE OF PAPERS.

51. The treatment of recurrent vascular ulcers of the cornea and phlyctenular pannus, by Dr. M'Gillivray, of Dundee.

52. Notes on a case of implantation of eyelash in the anterior chamber, by Dr. M'Gillivray, of Dundee.

53. Degrees of astigmatism, however low, when they annoy should be corrected, by Dr. Chisholm, of Baltimore, Md.

54. Two cases of spontaneous recurring intraocular hemorrhage, by Dr. Taylor, of Norwich.

55. A problem in neurology.—Peculiar iris-reaction with post-neuritic atrophy, by Dr. Gould, of Philadelphia, Pa.

56. The treatment of conical cornea by the thermo-cautery, by Dr. Emrys-Jones, of Manchester.

57. Operative procedure for distichiasis, by Dr. Germaix, of Algiers.

58. Pseudo-membranous conjunctivitis and its clinical forms in Egypt, by Dr. Sameh, of Cairo.

59. Javal's ophthalmometer employed for exophthalmometry and ophthalmostatometry, by Dr. Antonelli, of Naples.

60. Simple and combined sclerotomy, by Dr. De Wecker, of Paris.

61. On strabismus, especially strabismus of adolescents in relation to head-conformation, by Dr. Weiss, of Heidelberg.

ABSTRACTS OF PAPERS.

M'Gillivray (51) treats phlyctenular pannus and ulcers of the cornea with an ointment composed of atropine (alkaloid), yellow oxide of mercury and cocaine (alkaloid) in the proportion of grains j, ij, iij, respectively, with vaseline or lanoline ʒij, as a base. The ointment may be applied every two hours to commence with and gradually lessening the number of applications after healing has commenced.

M'Gillivray (52) also described a case in which a blow had been received on the right eye, nineteen days before the first examination. There was an irregularly-shaped leucoma situated horizontally and a little below the center of the cornea, and lying vertically in the anterior chamber was an eyelash which was adherent by one end to the center of the corneal cicatrix, while the other end rested on the anterior surface of the iris near the angle of the anterior chamber. Removal of the eyelash was refused and eighteen months after the injury there was no appearance of irritation and the vision was the same as that of the left eye.

Chisholm (53) protested against two statements of very general acceptance, namely, that irregular curvatures of the cornea, measuring less than 1.00 D., are not worthy of recognition, and that persons who have $V=\frac{20}{20}$ in each eye cannot have astigmatism. He had found that an astigmatism of 0.25 D. often causes serious eye disturbances and should then be corrected. "Head ache with eye-discomforts in young, healthy-looking people, who are not troubled during periods of eye-rest, means a low degree of annoying astigmatism."

Taylor (54) detailed two cases of that rare disease, spontaneous recurring intraocular hemorrhage. In an acute attack, he advised keeping the patient absolutely quiet, with head raised and ice applied to the eye and giving large doses of astringents and, possibly, when pulse is hard and full, applying three or four leeches near the outer canthus or, if the pulse has low tension, administering digitalis. Afterwards ferruginous aperients may be used and the eyes shaded and given prolonged rest.

Gould (55) reported a case of a girl twelve years old, who became totally blind from a most intense neuroretinitis in both eyes, followed by an absolute, white atrophy of both optic nerves and in which there was stabile mydriasis.

The most brilliant and concentrated light thrown suddenly or continuously on the pupil failed utterly to elicit iris-reaction or perception-response. But by seating the child before an open window, the street in front being illumined by sunlight or diffuse daylight, within half a minute or more, the pupils were found to be of normal size.

The parents had noticed the same effect when the child was out of doors. The contraction of the pupil was slow and so was the widening, when the patient's face was turned from the window. The mechanism by which this phenomenon was produced was a problem which suggested several queries.

Emrys-Jones (56) treats conical cornea with the thermo-cautery as follows: Fully dilate the pupil with atropine, anesthetize the cornea with a 4 per cent. solution of cocaine, and apply Paquelin's thermo-cautery with a moderately small point at red-heat, a little to the outside of the tip of the cone with great caution till the cornea is perforated, as shown by the escape of a thin spray of aqueous humor. The eye is then bandaged, the patient kept as much as possible on his back, and the pupil widely dilated. The burn becomes healed in seven to twenty-one days. In time the operation may be repeated if the cones do not disappear after the first operation. Most excellent results were reported.

Germaix (57) operates for distichiasis by a combination of several known procedures. He splits the edge of the lid sufficiently high to raise the anterior segment containing the ciliary roots till the cartilage beneath is exposed six to seven millimeters. A crescent-shaped piece of skin is also resected from the lid above, more or less wide, according to the laxity of the lids. Four sutures are then passed underneath and around the ciliary flap, so as to embrace it, and these are loosely drawn so as to raise and shorten the flap without strangling it, and are tied in front. The cicatrization between this flap and the superior part of the lid is allowed to take place without sutures by simple contact. The threads are removed on the third to the sixth day.

Sameh (58) contributes a long article on the clinical history and treatment of pseudo-membranous conjunctivitis as seen in Egypt.[1]

Antonelli (59) describes a new application of the ophthalmometer to the measurements of exophthalmos, whether pulsating or

1. There was a good deal of "threshing old straw" at the meeting, but the offering of this article was perhaps the most audacious effrontery of all. This paper had been read before at the French Ophthalmological Society, May 10, 1894, and may be found *verbatim* in the *Bulletins et Mémoires de la Société Francaise d'Ophthalmologie*, 1894, p. 318.

not, differences of level of the two eyes, the amplitude, frequence and direction of oscillations in nystagmus, etc., and various defects of position of the eyes. As an aid in determining some of these conditions, he has added an ingenious attachment to the ophthalmometer, which he calls the "ophthalmostatophantom." This he fully describes and illustrates.

De Wecker (60) presented an interesting paper on simple and combined sclerotomy for glaucoma. After relating something of the history of simple sclerotomy and operative iridodialysis, he says: "The ideal operation in glaucoma is evidently a large scleral incision combined with iridodialysis running the whole length of the cicatrix formed in the trabecular pericorneal tissue." This operation, which he terms "combined sclerotomy," he describes as follows: "After having put the eye fully under influence of eserine, and having instilled, twice only, and that immediately before the operation, one drop of a two per cent. solution of cocaine, I introduce my knife, which is six mm. wide up to the shoulder with which it is provided, at a point one mm. from the transparent border of the cornea, and I generally choose the point lying in the prolonged vertical diameter. Having allowed the aqueous humor to drain off very slowly so as to avoid any prolapse, I insert a very delicate pair of iridectomy forceps into the wound, of which the convex extremities of the blades are carefully rounded so as not to present the least roughness. As soon as the closed extremities are seen at a distance of two mm. from the corneal border, I open the forceps and seize the iris close to the periphery, and I very gently push back the part seized along the posterior surface of the cornea towards the center of the latter. A fairly free hemorrhage generally indicates the detachment, and, as the field of operation may be obscured by blood, one must bear in mind that the forceps, contrary to what one is accustomed to do, must be withdrawn with the blades *open*."

Weiss (61) has made extensive measurements of the face and head in patients affected with strabismus, and finds that these are different in cases of the convergent form from those of the divergent.

The head-diameters, "temporal, aural, greatest zygomatic," are taken, and the difference between the inner and outer orbital edges, the angle of inclination of the orbital entrance, the pupillary distance, the distance of the "corneal vertex" from the external orbital margin are determined. In divergent strabismus he found that the planes of the sides of the face are directed more exactly forwards (parallel), while in convergent strabismus these planes converge, anteriorly, more or less. He also found that in children with hypermetropia the axes of the orbits are directed forwards more exactly parallel, while in myopic children they diverge. He believes that these conformations, as determined by the measurements which he describes, have a practical bearing upon the treatment of strabismus in children, and should always be taken into consideration.

